# Shoulder Intra-Articular Temperature Is Higher In Patients With Small Rotator Cuff Tears Compared With Patients Who Have Larger Tears

**DOI:** 10.1016/j.asmr.2023.100813

**Published:** 2023-10-24

**Authors:** Stefano Gumina, Marco Rionero, Jacopo Preziosi Standoli, Matteo Cantore, Vittorio Candela

**Affiliations:** a Department of Anatomical, Histological, Forensic Medicine and Orthopaedics Sciences, Sapienza University of Rome, Rome, Italy; b Istituto Clinico Ortopedico Traumatologico (ICOT), Latina, Italy; c Sapienza University, Sant'Andrea Hospital, Rome, Italy

## Abstract

**Purpose:**

The purpose of this study was to determine whether the intra-articular temperature of the shoulder correlates with the size of the tendon tear in patients with rotator cuff tears (RCTs).

**Methods:**

The shoulder intra-articular temperature of 75 consecutive (32 female, 43 male; mean age 61.12; standard deviation = 7.10) patients who underwent arthroscopic rotator cuff repair was measured with a digital thermometer, at first in 2 points (biceps anchor and glenoid labrum) during dry arthroscopy, followed by a third measurement during wet arthroscopy. A fourth measurement, represented by the patient’s axillary body temperature, was taken upon admission. The RCTs were classified during surgery according to the Southern California Orthopedic Institute classification system as small, large, and massive. Data were submitted for statistical analysis.

**Results:**

The intra-articular temperature differs in patients with different-sized RCTs regardless of the location of the thermometer. A significantly higher temperature was found in patients with small RCTs (36.2°C ± 0.57°C) (*P* < .01). When the in-flow of the arthroscopic fluid was opened, the temperature dropped to an average of 24.5°C.

**Conclusions:**

The shoulder intra-articular temperature was significantly associated with RCT size. A significantly higher temperature was found in small RCTs. No correlation was found between age and sex, age and RCT size, sex and RCT size, or sex and temperature.

**Clinical Relevance:**

An early diagnosis and treatment of RCTs may avoid further degeneration and damage of the tendon caused by the increased temperature.

A higher-than-normal intra-articular temperature has been found in the osteoarthritic knee joint.^1^, ^2^, ^3^ An increase in intra-articular temperature is usually attributed to hyperemia.^4^, ^5^, ^6^ Elevated temperatures have been shown to cause severe damage to collagen fibers of the extracellular matrix through an irreversible transformation of the normal linear structure into a more random and disorganized one.^1^^,^^7^, ^8^, ^9^, ^10^ Harris and McCoskery^1^ observed that in rheumatoid knee joints, which showed a mean intra-articular temperature of 36°C, collagenolysis was fourfold greater than in healthy knee joints (33°C). It has been demonstrated that at 37°C type I collagen taken from human lungs forms random coils within 2 to 3 days.^8^ At the same temperature, type I collagen from rat tail tendon is even less stable.^8^

It has been shown that rotator cuff tearing is characterized by intra-articular inflammation and subacromial bursitis and that an inverse correlation exists between the severity of the inflammation and the tendon tear size.^11^, ^12^, ^13^, ^14^, ^15^, ^16^ The purpose of this study was to determine whether the intra-articular temperature of the shoulder correlates with the size of the tendon tear in patients with rotator cuff tears (RCTs). We hypothesized that small rotator cuff tendon tears would have higher shoulder intra-articular temperatures than large and massive tears.

## Methods

A prospective analysis of 87 consecutive patients (38 females and 49 males) who underwent arthroscopic repair of a full-thickness rotator cuff tear as diagnosed by clinical examination and magnetic resonance imaging between January 2021 and January 2022 was performed. All patients were treated in the same hospital.

Patients with concomitant calcific tendinitis, shoulder instability, frozen shoulder, history of shoulder surgery, trauma or inflammatory disease were excluded. Demographic data including sex, age, body mass index, and side of the shoulder were collected. The Southern California Orthopaedic Institute classification of complete rotator cuff tears was used to identify 3 different groups^17^:

1. A small, complete tear, such as a puncture wound or a tear (usually <2 cm), that still encompasses only 1 rotator cuff tendon, with no retraction of the torn ends
2. A large, complete tear involving an entire tendon, with minimal retraction of the torn edge, usually 3 to 4 cm
3. A massive rotator cuff tear involving >2 rotator cuff tendons, frequently with associated retraction and scarring of the remaining tendon ends, and often an L-shaped tear that is frequently irreparable

**Fig 1:**
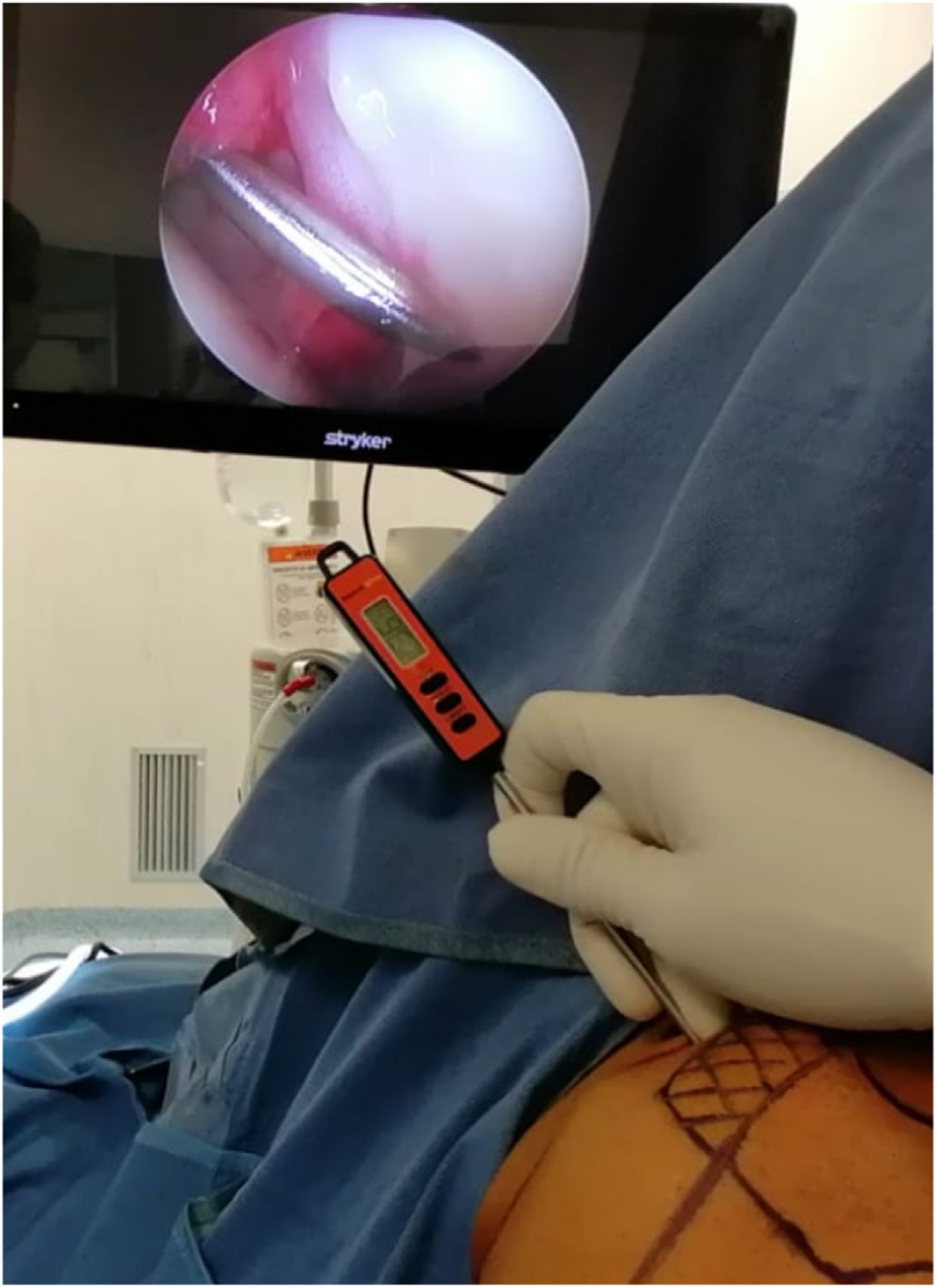
Left shoulder; measurement of the glenoid labrum temperature from the anterior portal.

During arthroscopic repairs, the temperature of the operating room was kept stable at 21°C. All patients underwent an interscalene block in an holding area before being transferred into the operating room where the general anesthesia was then performed. All patients underwent operation in the beach-chair position. A warm blanket was used in all cases to prevent hypothermia. Neither the shoulder joint nor the arthroscopic portals were injected with local anesthetic. The arthroscope was inserted through the posterior portal and positioned near the posterior ridge of the glenoid with the illumination set at the minimum level so that the heat generated by the light source would not alter the measurements. A digital thermometer (accuracy of ± 0.2°C [range: −50°C to 300°C) was then inserted through the anterior portal during dry arthroscopy (Fig 1) with the light source at the minimum, and the temperature was taken in 2 different points: under the biceps anchor (BAT) and at the glenoid labrum (GLT) at the 4 o'clock position in a right-side shoulder (8 o’clock position in a left shoulder). The articular surfaces were not touched. A third measurement was taken at the glenoid labrum after the inflow of the arthroscopic fluid was allowed. All 3 intra-articular measurements were performed by 2 of the surgeons (S.G. or M.R.) and confirmed by a third surgeon (V.C.). The fluid bags were stored at 18°C. The fourth measurement was represented by the axillary body temperature measured in degrees Celsius with a second digital termomether (like the one used in the operating room) the same day of surgery in a temperature-controlled room ∼20 minutes after patient’s admission by 1 of the authors (M.C.). The whole procedure was carried out by the same surgical team each time. All measurements were taken with the same termomether, which was calibrated and sterilized between surgeries. Patients signed an informed consent form as according to the Declaration of Helsinki, and the study was approved by the local ethical committee.

### Statistical Analysis

The Shapiro-Wilk test was used to assess the normal data distribution. Categorical variables were calculated using frequencies and proportions whereas continuous data has been estimated by means, standard deviations, and ranges.

One-way analysis of variance testing or the Kruskal-Wallis test was used to analyze differences among 3 groups. The χ^2^ test was conducted for statistical analysis concerning categorical data when appropriate. Significant levels for multiple comparisons were adjusted using the Bonferroni procedure. Calculated *P* values were 2-tailed, a *P*-value <.05 was considered significant, and the range of confidence interval was 95% when appropriate. Statistical analysis was performed using JASP Team (2020). JASP (Version 0.14.1) (https://jasp-stats.org/).

An a priori calculation of sample size was done using g∗power 3.1.9.4 software (Heinrich-Heine-University, Dusseldorf, Germany). According to the analysis, at least 54 patients would be required (18 patients for each group), assuming a 2-tailed α value = 0.05 (sensitivity = 95%) and a β value = 0.95 (with a study power of 95%).

## Results

The original study group was composed of 87 patients. After excluding patients with concomitant calcific tendinitis, shoulder instability, frozen shoulder, history of shoulder surgery, trauma, or inflammatory disease, the final study group was composed of 75 patients (32 female; 43 male) with different sizes of RCT. A total of 12 patients were excluded (4 patients presented glenohumeral instability, 3 patients were treated for calcific tendinitis, 2 patients suffered from frozen shoulder syndrome, 2 patients were diagnosed with traumatic RCT, and 1 patient had a recurrence of RCT). The baseline characteristics of the studied group are reported in Table 1. No statistical correlations have been found between age and sex (*P* = .22), age and RCTs sizes (*P* = .56), or gender and RCTs sizes (*P* = .39).

**Table 1:** Baseline Characteristics of the Studied Group according to Sex and RCTs

	Females	Males	Small RCT	Large RCT	Massive RCT	Total
No.	32 (42.7%)	43 (57.3%)	29 (38.7%)	22 (29.3%)	24 (32%)	75
Age (y), mean (SD)	62 (7.4)	60 (6.7)	60 (6.8)	61 (5.8)	62 (8.4)	61
Range	48-72	45-71	45-72	48-70	47-71	45-72

RCT, rotator cuff tear.

The registered temperatures are summarized in Table 2. No statistical correlation has been found between sex and all 3 temperatures (body temperature, *P* = .36; BAT, *P* = .42; GLT, *P* = .54) or body mass index and body temperature (*P* = 0.08).

**Table 2:** The Measured Temperatures (in Degrees Celsius) According to Different-Sized RCTs

	Body Temperature			Biceps Anchor Temperature			Glenoid Labrum Temperature		
	Small RCT	Large RCT	Massive RCT	Small RCT	Large RCT	Massive RCT	Small RCT	Large RCT	Massive RCT
Mean	36.0°	36.2°	35.9°	36.2°	35.9°	35.7°	36.1°	35.8°	35.6°
SD	0.4	0.4	0.4	0.6	0.6	0.6	0.6	0.6	0.5
Range	35°-36.6°	35.5°-36.7°	35°-36.6°	34.3°-37.3°	34.9°-37°	34.6°-37°	34.6°-37.3°	34.4°-37°	34.9°-37.2°

RCT, rotator cuff tear; SD, standard deviation.

**Fig 2:**
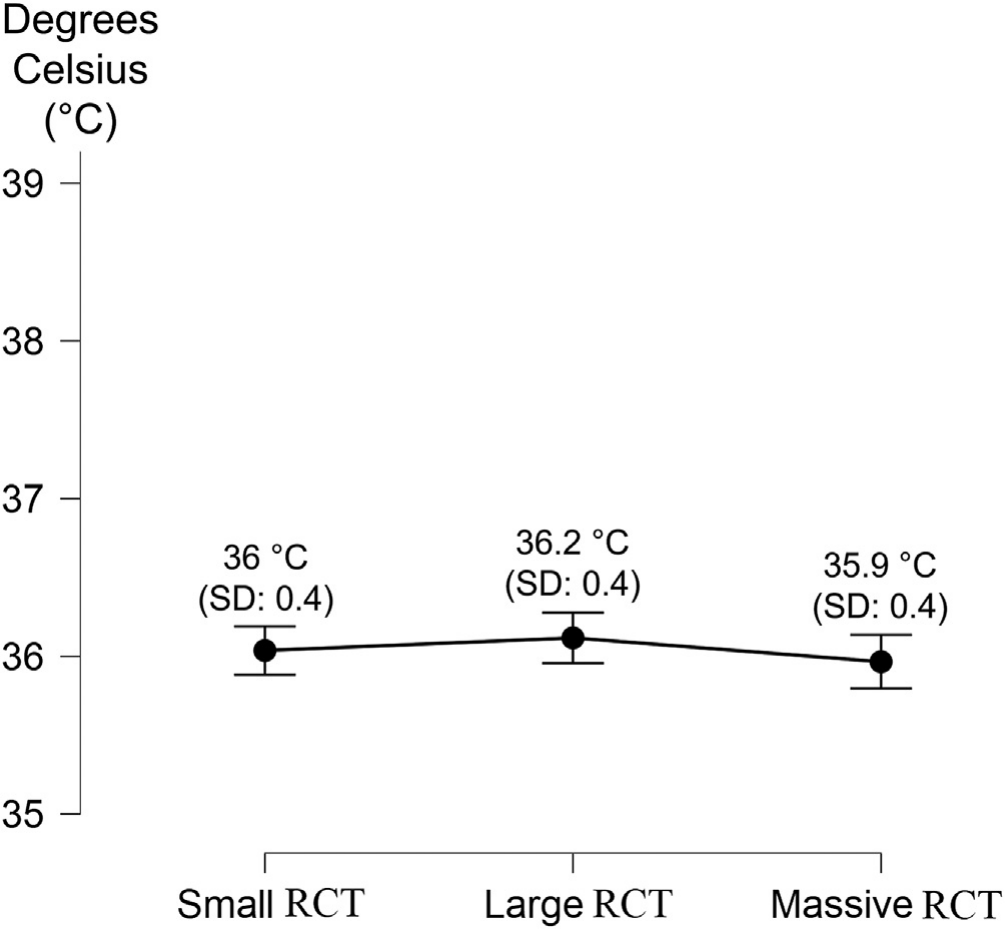
The graph reports the mean plus standard deviation of the measured body temperature according to the rotator cuff tear (RCT) size. The temperature is shown on the Y axis, whereas the RCT type is shown on the X axis.

Data according to body temperature and RCTs sizes are reported in Figure 2. No statistical correlation has been found (*P* = .43).

**Fig 3:**
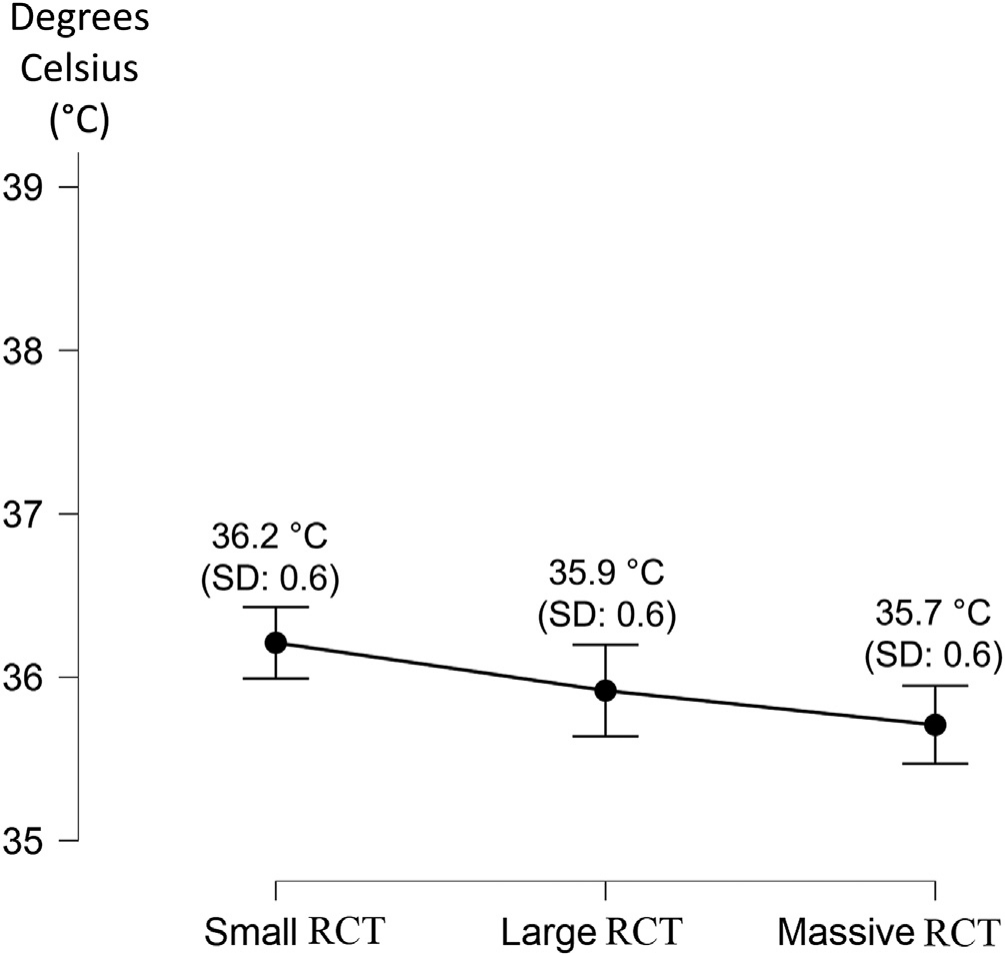
The graph reports the mean plus standard deviation of the measured biceps anchor temperature according to the rotator cuff tear (RCT) size. The temperature is shown on the Y axis, whereas the RCT type is shown on the X axis.

**Fig 4:**
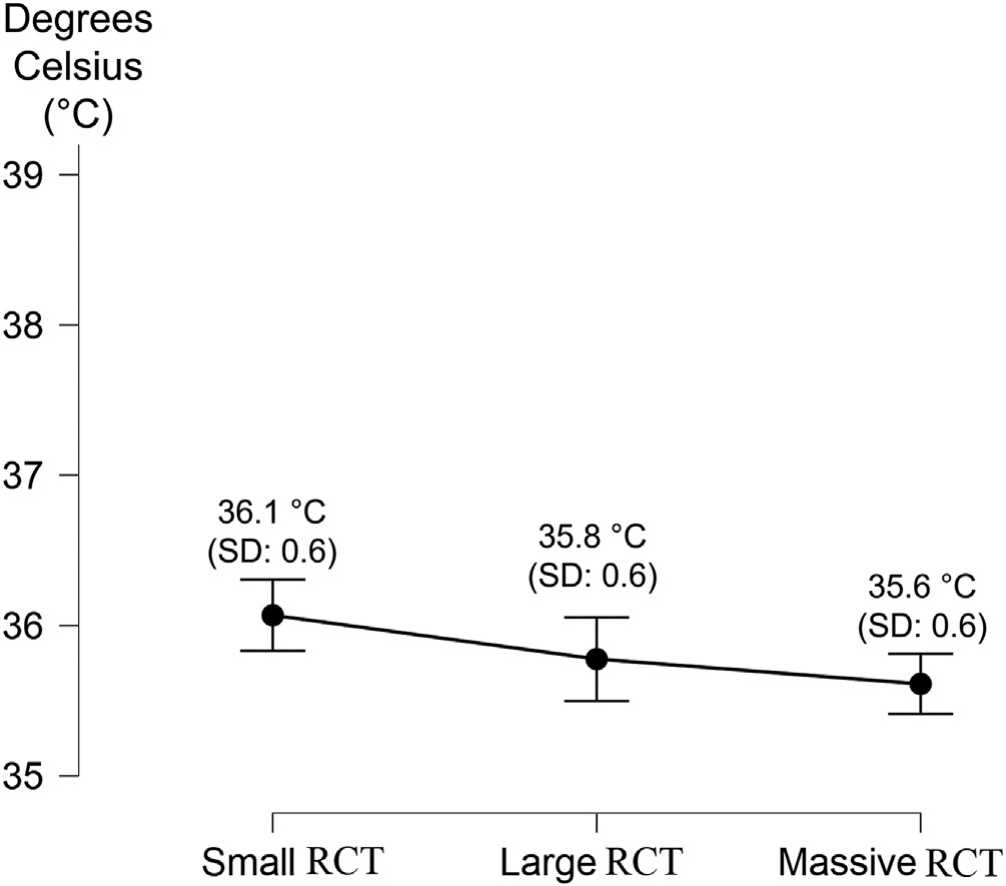
The graph reports the mean plus standard deviation of the measured glenoid labrum temperature according to the rotator cuff tear (RCT) size. The temperature is shown on the Y axis, whereas the RCT type is shown on the X axis.

The BAT and GLT temperatures differ in patients with different sized RCTs; in particular, BAT and GLT were significantly higher in patients with small RCTs (*P* < .01) (Figs 3 and 4). When the in-flow of the arthroscopic fluid was opened, the mean temperature registered at the biceps anchor was 24.51°C ± 1.79°C.

## Discussion

The most important findings of this study are that the intra-articular temperature differs in patients with different-sized RCTs; in particular, BAT and GLT were significantly higher in patients with small RCTs (*P* < .01). A total of 29 patients had a small tear, 22 a large tear, and 24 a massive tear. The mean body temperature in the 3 groups was 36°C, 36.2°C, and 35.9°C, respectively. The BATs were 36.2°C, 35.9°C, and 35.7°C, respectively. The GLTs were 36.1°C, 35.8°C, and 35.6°C, respectively.

Studies have shown that in healthy knee joints the intra-articular temperature ranges from 30.5°C to 33°C.^2^^,^^3^^,^^18^ Haimovici^19^ and Harris and McCroskery^1^ analyzed the same parameter and found a temperature of 32.8°C and 33°C, respectively. Kim et al.^20^ measured the knee skin and intra-articular temperature of 18 healthy volunteers and the effect of cold air application. They found that the baseline intra-articular temperature was of 33.9°C ± 1.2°C. Becher et al.^21^ observed that in young athletes who practiced jogging and skiing, the knee intra-articular temperature was on average of 31.4°C (range, 29.7°C-34.3°C) and 32.2°C (range, 29.7°C-32.3°C), respectively, although without a statistically significant difference between the 2 groups. Two considerations can be drawn from these studies: first that current knowledge about intra-articular temperature comes from studies conducted principally on the knee joint and second that the intra-articular temperature fluctuates within wide normal limits.

Studies have shown that intra-articular temperature is influenced by active weight bearing motions,^2^ radiant heat,^2^ high-frequency electrical energy,^2^ application of paraffine to the surface,^2^ application of hot or cold packs,^2^^,^^3^^,^^20^^,^^22^^,^^23^ and high-intensity activity.^21^ Although to a lesser extent, passive motion,^2^ room temperature,^18^ low outside temperature,^21^ and hydrotherapy^21^ are also capable of modifying the intra-articular temperature.

Different authors have documented a close correlation between the internal joint temperature and the clinical activity in patients suffering from arthritic diseases.^1^, ^2^, ^3^ It has been observed that joints affected by active synovitis show intra-articular temperatures between 34°C and 37.6°C.^2^^,^^3^^,^^18^ The temperature rise is attributed to tissue hypervascularization.^9^ In this regard, many studies have confirmed an increase in synovial and subacromial vascularity in shoulders with rotator cuff tear, as well as a correlation between RCTs and macroscopic signs of glenohumeral synovitis.^24^, ^25^, ^26^, ^27^ Longo et al.,^28^ using a semiquantitative assessment of the tendinous lesions, observed a markedly abnormal increase in vascularity in 60% of the supraspinatus tendons with a tear, whereas none (0%) of the healthy tendons (controls) showed changes in vascularization.

Close to the margin of a cuff tear, fibrocytes and newly formed vessels, bordered by intumescent endothelial cells, were detected.^29^^,^^30^ Many different growth factors, including the vascular endothelial growth factor, are markedly upregulated after tendon injury and have an active role in the multiple phases of the healing process.^31^^,^^32^ Furthermore, angiopoietin 1, which participates in vessel maturation and stabilization, is upregulated in synovial fluids and serum of patients with an RCT.^33^ In addition, the expression of hypoxia inducible factor and vascular endothelial growth factor, important inducers of neoangiogenesis, were higher in full-thickness medium-sized RCTs compared with healthy tissues.^34^ Finally, the nuclear factor-kB, which regulates apoptosis and stimulates neoangiogenesis, was found on RCT margins and in subacromial bursal tissue.^29^ Because high temperatures may be responsible for severe damage to the collagen fibers of the extracellular matrix through an irreversible transformation of the normal linear structure into a more random and disorganized one, the aim of this study was to investigate whether patients with different sizes of cuff tear also showed a pathological increase in temperature, which, together with mechanical^35^, ^36^, ^37^ and cellular^38^ conditions, could facilitate the progression of tissue disorganization and, consequently, tear size.

Both intra-articular temperatures measured during dry arthroscopy were higher than that reported in the literature for healthy knees^3^^,^^20^^,^^21^ but are similar to those recorded in arthritic knees.^2^^,^^3^^,^^18^ Furthermore, the biceps tendon and the GLTs in patients with a small cuff tear is significantly higher than that found in shoulders of patients with a large or massive tear. These data indicate that hyperemia, after the inflammatory process involving tendons and subacromial bursa, is able to raise the shoulder intra-articular temperature in patients with a cuff tear. Because shoulders with a small tear are significantly more inflamed than those with a massive tear,^11^ it can be inferred that as the size of the tear progresses there is a decrease in tissue inflammation/hyperemia and consequently a progressive decrease in intra-articular temperature.

By accepting the hypothesis that severe damage to collagen fibers of the extracellular matrix is caused by an increase in intra-articular temperature, it is equally conceivable that the increase in intra-articular temperature, registered in shoulders of patients with a small cuff tear, may contribute to the progression of tear size.

Recently, Gumina et al.^38^ suggested early operation on young patients with small tears because nuclear stability and vitality of tenocytes close to tear edges decreases with increasing tear size. This study supports this suggestion because a small tear may progress in size, and the increase in intra-articular temperature could also facilitate tissue degeneration progress.

Finally, it was observed that when the inflow of the arthroscopic fluid was allowed, the joint temperature decreased by about 11°C. The clinical implications of this drastic and sudden intra-articular variation in temperature are unknown because, as observed by Zaffagnini et al.,^39^ it is difficult to determine the recovery time of the joint temperature and consequently the real effect on joint tissue.

### Limitations

This study is not without limitations. Although the RCT was the focus, temperatures were not taken in the bursa where inflammation would be expected. The probe was not placed on the torn tendon but at the base of the biceps tendon and near the glenoid labrum. This was done to ensure that temperature measurement was reproducible. However, these temperature sites may not reflect inflammation associated with rotator cuff pathology. Another limitation is that no control data from patients with an intact rotator cuff was obtained for comparison.

## Conclusions

The shoulder intra-articular temperature was significantly associated with RCT size. A significantly higher temperature was found in small RCTs. No correlation was found between age and sex, age and RCTs sizes, sex and RCTs sizes, or sex and temperatures.

## Supplementary Data

ICMJE author disclosure forms
